# Supplementary material for: The interactome of intact mitochondria by cross-linking mass spectrometry provides evidence for coexisting respiratory supercomplexes
Source: Mol Cell Proteomics. 2017 Dec 8;17(2):216–32. doi: 10.1074/mcp.RA117.000470 (PMC5795388; doi:10.1074/mcp.RA117.000470)
Supplement: Supplemental Data [file supp_RA117.000470_133922_0_supp_23978_fzffwf.zip › spectra_annotation/mito_spectra_annotation/134-1-6-1-16-1.pdf]

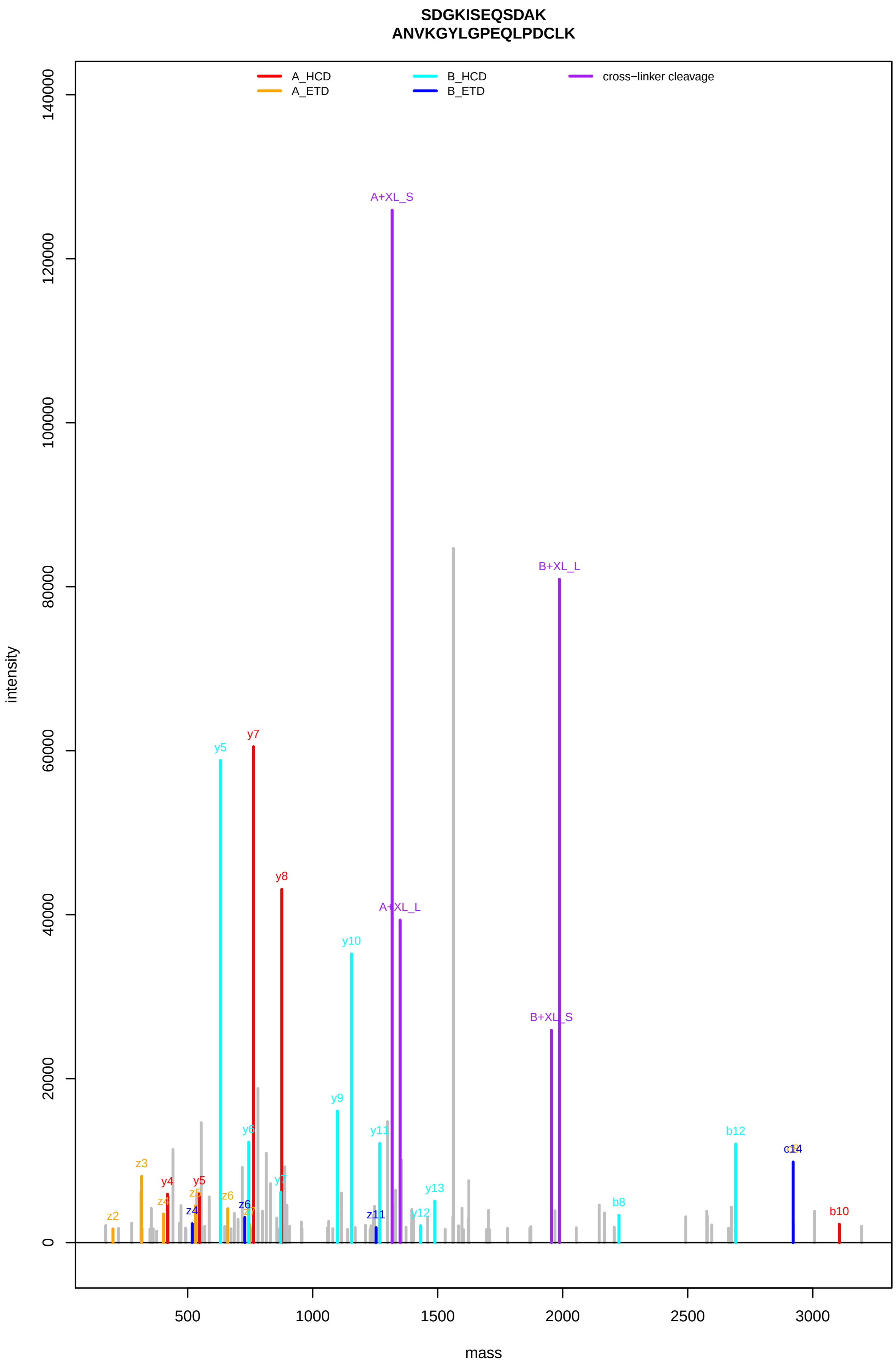
SDGKISEQSDAK
ANVKGYLGPEQLPDCLK
A_HCD
A_ETD
B_HCD
B_ETD
cross−linker cleavage
A+XL_S
B+XL_L
A+XL_L
B+XL_S
intensity
mass
y5
y7
y8
y10
y9
y11
y13
y12
b12
b8
c14
b10
z3
z2
y4
z4
y5
z5
z6
z4
z6
y6
y7
z11
0
20000
40000
60000
80000
100000
120000
140000
500
1000
1500
2000
2500
3000

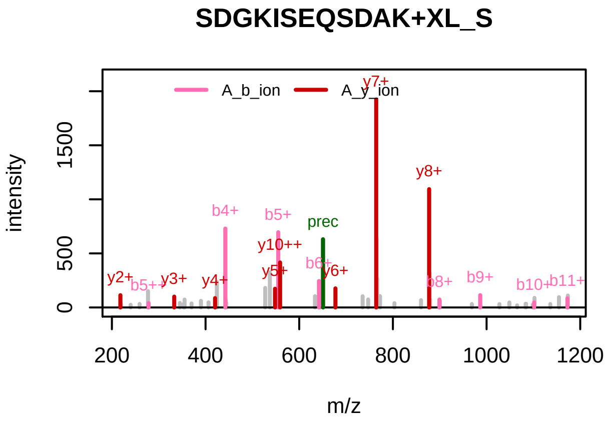
SDGKISEQSDAK+XL_S
A_b_ion
A_y_ion
intensity
m/z
y7+
y8+
b4+
b5+
prec
y10++
y5+
b6+
y6+
y2+
b5++
y3+
y4+
b8+
b9+
b10+
b11+
0
500
1500
200
400
600
800
1000
1200

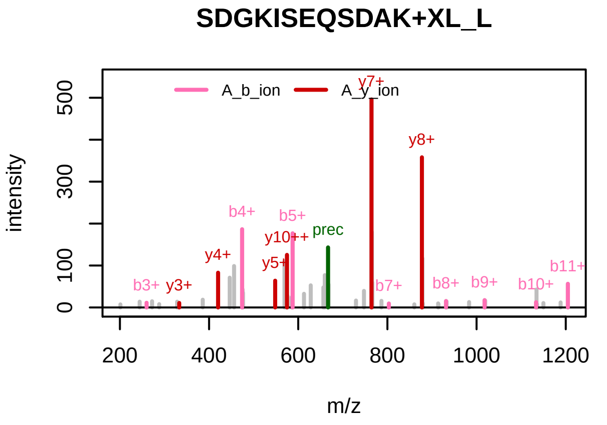
SDGKISEQSDAK+XL_L
A_b_ion
A_y_ion
intensity
m/z
y7+
y8+
b4+
b5+
y10++
prec
y4+
y5+
b3+
y3+
b7+
b8+
b9+
b10+
b11+
0
100
300
500
200
400
600
800
1000
1200

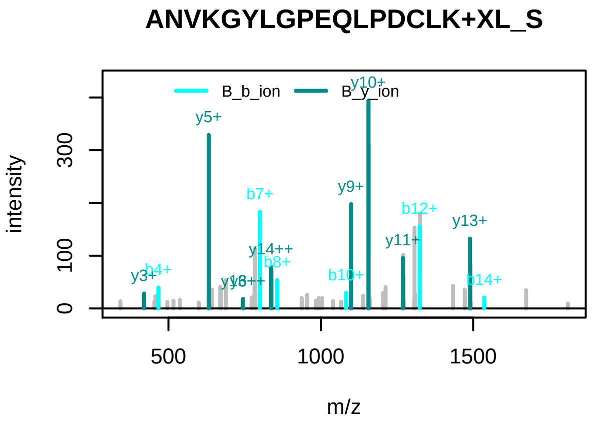
ANVKGYLGPEQLPDCLK+XL_S
B_b_ion
B_y_ion
intensity
m/z
y10+
y5+
b7+
y9+
b12+
y13+
y11+
y14++
b8+
y3+
b4+
b10+
b14+
0
100
300
500
1000
1500

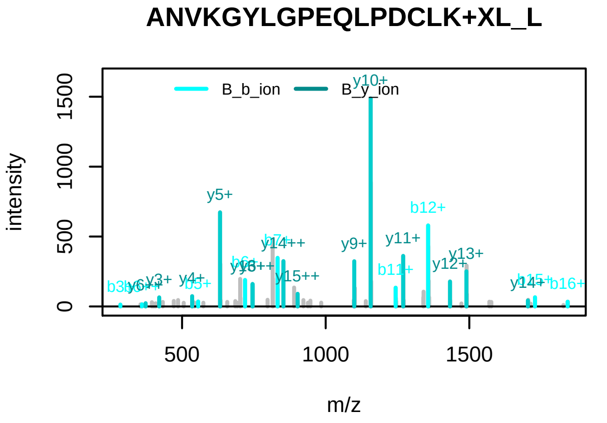
ANVKGYLGPEQLPDCLK+XL_L
B_b_ion
B_y_ion
intensity
m/z
y10+
y5+
b12+
y14++
y9+
y11+
y13+
y12+
b11+
y15++
y3+
y4+
b3+
y6++
b5+
y14+
b15+
b16+
0
500
1000
1500
500
1000
1500
